# Visualization of the intracavitary blood flow in systemic ventricles of Fontan patients by contrast echocardiography using particle image velocimetry

**DOI:** 10.1186/1476-7120-10-5

**Published:** 2012-02-20

**Authors:** Konstantinos Lampropoulos, Werner Budts, Alexander Van de Bruaene, Els Troost, Joost P van Melle

**Affiliations:** 1 Department of Cardiology, University Hospitals Leuven, Leuven, Belgium; 2 Department of Cardiology, Polyclinic General Hospital of Athens, Athens, Greece; 3 Department of Cardiology, University Medical Center Groningen, University of Groningen, Groningen, The Netherlands; 4 Congenital and structural cardiology, University Hospitals Leuven, Herestraat 49, 3000 Leuven, Belgium

**Keywords:** Fontan patients, Vortex, Contrast echocardiography, Particle image velocimetry

## Abstract

**Background:**

Flow patterns in univentricular hearts may have clinical value. Therefore, it is our objective to asses and characterize vortex flow patterns with Fontan circulation in comparison with healthy controls.

**Methods:**

Twenty-three patients (8 Fontan and 15 normal patients) underwent echocardiography with intravenous contrast agent (Sonovue^®^) administration. Dedicated software was used to perform particle image velocimetry (PIV) and to visualize intracavitary flow in the systemic ventricles of the patients. Vortex parameters including vortex depth, length, width, and sphericity index were measured. Vortex pulsatility parameters including relative strength, vortex relative strength, and vortex pulsation correlation were also measured.

**Results:**

The data from this study show that it is feasible to perform particle velocimetry in Fontan patients. Vortex length (VL) was significantly lower (0.51 ± 0.09 vs 0.65 ± 0.12, *P *= 0.010) and vortex width (VW) (0.32 ± 0.06 vs 0.27 ± 0.04, *p *= 0.014), vortex pulsation correlation (VPC) (0.26 ± 0.25 vs -0.22 ± 0.87, *p *= 0.05) were significantly higher in Fontan patients. Sphericity index (SI) (1.66 ± 0.48 vs 2.42 ± 0.62, *p *= 0.005), relative strength (RS) (0.77 ± 0.33 vs 1.90 ± 0.47, *p *= 0.0001), vortex relative strength (VRS) (0.18 ± 0.13 vs 0.43 ± 0.14, *p *= 0.0001) were significantly lower in the Fontan patients group.

**Conclusions:**

PIV using contrast echocardiography is feasible in Fontan patients. Fontan patients had aberrant flow patterns as compared to normal hearts in terms of position, shape and sphericity of the main vortices. The vortex from the Fontan group was consistently shorter, wider and rounder than in controls. Whether vortex characteristics are related with clinical outcome is subject to further investigation.

## Introduction

Particle image velocimetry is a new technique of determining the velocity and the direction of fluid streams by analyzing the change in position of small particles that drift with the fluid. With the recent development of echocardiographic technology, it is now possible to apply this approach to contrast-enhanced echocardiographic imaging [1-3].

The growing knowledge about the structure and function of the ventricle [4] was of high interest to us in the context of ventricular vortex development in the Fontan patients. A vortex is a mass of fluid with a whirling or circular motion, thus containing kinetic energy. It is supposed to increase cardiac efficiency by maintaining the momentum of the inflowing blood in diastole and, thus, facilitating systolic ejection of blood into the left ventricular outflow tract[5]. The demonstration of diastolic vortex formation in normal human hearts and their distortion by valve surgery [6] led to our study objective to what extent blood flow patterns in congenitally abnormal, functionally univentricular hearts of Fontan patients are different from normal ones.

This study aimed at assessing vortex flow patterns in patients with Fontan circulation in comparison with healthy controls.

## Methods

### Study population

A total of 23 patients were enrolled in this study: 8 Fontan [age 31.5 ± 12 years (mean ± standard error of the mean, SEΜ); 7 women] and 15 controls [age 46 ± 10 years (mean ± standard error of the mean, SEΜ); 6 women]. All patients were in a stable clinical condition, without overt signs or symptoms of heart failure, classified as functional class NYHA I-II. Oxygen saturations of all patients were above 96% indicating the absence of major right-to-left shunting. The study protocol was approved by the ethics committee of our institution. All patients gave written informed consent prior to the examinations. Demographic and clinical characteristics of our study population are summarized in Tables 1 and 2.

**Table 1:** Demographic and clinical characteristics of our study population

Variables	Fontan patients	Controls	*p *value
**Age, yrs**	31.5 ± 12	46 ± 10	0.243

**Women/Men**	6/2	6/9	0.252

**LVEDd**	51.8 ± 8,8	47.5 ± 0.7	0.210

**LVESd**	37.1 ± 8.2	32.5 ± 2.1	0.199

**LAd**	40.7 ± 13.9	37.5 ± 0.7	0.334

**Systemic FS%**	29 ± 4.4	35 ± 5	0.071

**Table 2:** Clinical features of the 8 Fontan patients

Patient	Age (years)	Sex	Defects
**1**	22	Male	TA, VSD, subvalvular PS, left SVC

**2**	55	Female	Ebstein's anomaly, VSD, TS

**3**	37	Female	TA, VSD

**4**	29	Male	DILV, VSD, pulmonary atresia

**5**	19	Female	TA, VSD, subvalvular PS

**6**	23	Female	cc-TGA, TA, ASD, PS, VSD, left SVC

**7**	35	Female	DORV, TGA, PS, abnormal pulmonary venous connection

**8**	42	Female	TA, VSD, ASD

### Imaging

To visualize intracavitary flow, a 0.2 ml of intravenous contrast (Sonovue^®^) was injected intravenously as a bolus. Images in the apical 4 chamber views plane were obtained and digitally stored using a Sequoia C512 (Siemens, Mountain View, USA) equipped with a 3.5 MHz transducer. Data were analyzed off-line with dedicated research software (Omega Flow, Siemens, Mountain View, USA) capable of tracking contrast enhanced blood flow and visualizing and quantifying blood flow patterns as described recently [4].

### Quantitative vortex flow parameters

The process of vortex formation It is related to the difference in velocity between the high-speed inflow jet after mitral valve opening and the surrounding still fluid in the left ventricle (LV). The shear layer between the moving and the still part of the blood promotes natural swirling of flow inside the ventricle, leading to the vortex formation

A vortex is a mass of fluid whose elements are moving in nearly circular pathlines about a common axis. Vortices are to be distinguished from vorticity, which is the local rate of rotation of infinitesimal fluid elements about their own axes.

The parameter vorticity, describing the curling of the blood flow was colour coded and displayed over the entire cardiac cycle. For the evaluation of vortex morphology and characteristics, we used the parameters as was proposed by Hong et al.[3] In brief, we measured vortex depth (VD), vortex length (VL) and vortex width (VW) indicating the shape of vortex. VL was measured using major-axis length of vortex relative to systemic ventricle length, VW was measured using minor-axis length of vortex relative to systemic ventricle length, and VD represents vertical position of the center of vortex relative to systemic ventricular long axis. A vortex sphericity index (SI) was calculated by VL and VW, as VL/VW ratio.

Changes in regional vorticity can be used to estimate energy dissipation in the fluid. A vortex loses kinetic energy because of fluid viscosity, when a large, and steady vortex is converted in the bloodstream into smaller, rapidly changing vortices. The pulsatility map showed red colour if there is strong vortex. For the evaluation of energy dissipation (pulsatility) of left ventricle vortex, we assessed 3 pulsatility parameters including relative strength (RS), vortex relative strength (VRS), and vortex pulsation correlation (VPC) of left ventricle vortex.

The RS represents the strength of the pulsatile component of vorticity with respect to the average vorticity in the whole left ventricle. The VRS represents the same ratio accounting for the pulsatile vorticity of vortex only instead of the entire left ventricle. The VPC is the correlation between steady and pulsatile vorticity in the vortex, normalized with the vortex strength and area to make a dimensionless parameter.

RS and VRS can be calculated with a mathematic definition as follows [6]:

$$VRS=\frac{\underset{vortex}{\int }\omega_{1}\left(x,y\right)dxdy}{\underset{vortex}{\int }\omega_{0}\left(x,y\right)dxdy},$$

$$RS=\frac{\underset{LV}{\int }\omega_{1}\left(x,y\right)dxdy}{\underset{vortex}{\int }\omega_{0}\left(x,y\right)dxdy}$$

where ω_0 _represents the steady and ω_1 _represents the pulsatile component (first harmonic) of regional vorticity.

The VPC can be calculated with a different mathematic definition as follows [6]:

$VPC=\frac{Avortex\underset{vortex}{\int }\omega_{0}\left(x,y\right)\omega_{1}\left(x,y\right)dxdy}{\left(\underset{vortex}{\int }\omega_{0}\left(x,y\right)dxdy\right)\begin{array}{c} {}^{2} \end{array}},$where ω_0 _represents again the steady and ω_1 _represents the pulsatile component (first harmonic) of regional vorticity and A_vortex _represents the vortex area.

Two double-blinded examiners repeatedly performed the measurements on 10 randomly selected patients for interobserver analysis. The second observer repeated the measurements 2 months after the first time. Case sequence was randomly arranged each time.

### Statistical analysis

Continuous variables were presented as mean and standard deviation and were compared using the independent Student *t *test. Comparison of categorical variables was made by the chi-square test. The assessment of intraobserver and interobserver variability was performed by two independent observers or within an observer for quantitative vortex parameters. The agreement between the two measurements was expressed using the 95% confidence interval and determined as the mean of the differences +/-1.96SD [7].

A p value < 0.05 was considered statistically significant. All statistical analyses were performed using the SSPS statistical package (SSPS version 18.0, Inc., Chicago, Illinois)

## Results

### Clinical data

Clinical characteristics of the study population are shown in Tables 1 and 2. Mean age was 46 ± 10 years for the control group. Of the 8 patients with Fontan circulation (mean age 31.5 ± 12 years), 2 patients (25%) were male. There was no significant difference in age and gender between the controls and Fontan patients groups. The right ventricular systolic function was estimated visually by an experienced echocardiogram while the left ventricular systolic function was calculated by Volumetric Biplane Simpsons' (FS: 29 ± 4.4 vs. 35 ± 5%, p = 0.07), the systemic ventricular end-diastolic dimension (51.8 ± 8.8 vs. 47.5 ± 0.7, *p *= 0.2), systemic ventricular systolic dimension (37.1 ± 8.2 vs. 32.5 ± 2.1, *p *= 0.1), and left atrial dimension (42.7 ± 13.8 vs. 37.5 ± 0.7, *p *= 0.2) were similar in both groups.

### Characterization of systemic ventricular vortex flow

**Figure 1:**
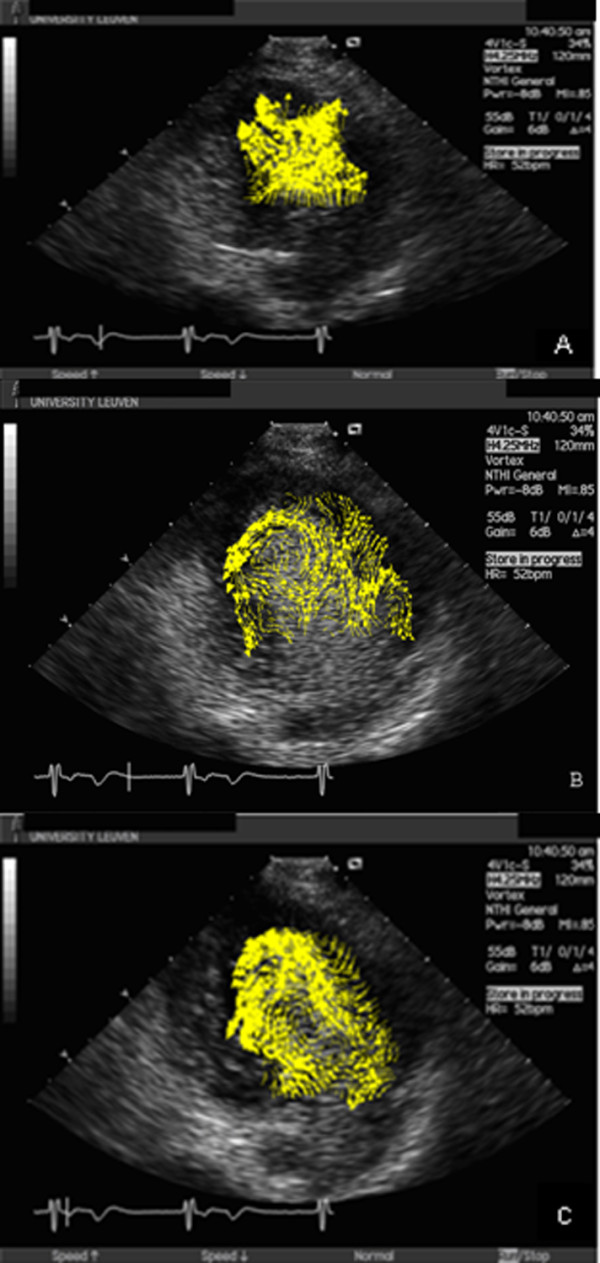
**Sequence analysis of systemic ventricular flow during systole and diastole in Fontan patients and controls**. The vortex from the Fontan group was consistently shorter, wider and rounder than in controls. The vortices were located at the centre of the left ventricle throughout diastole and systole and did not redirect flow in a coherent, sequential fashion as in controls. The location, shape and sphericity of the main vortices differ clearly from controls in all cardiac cycle [early diastole(A), late diastole(B), ejection (C)].

**Figure 2:**
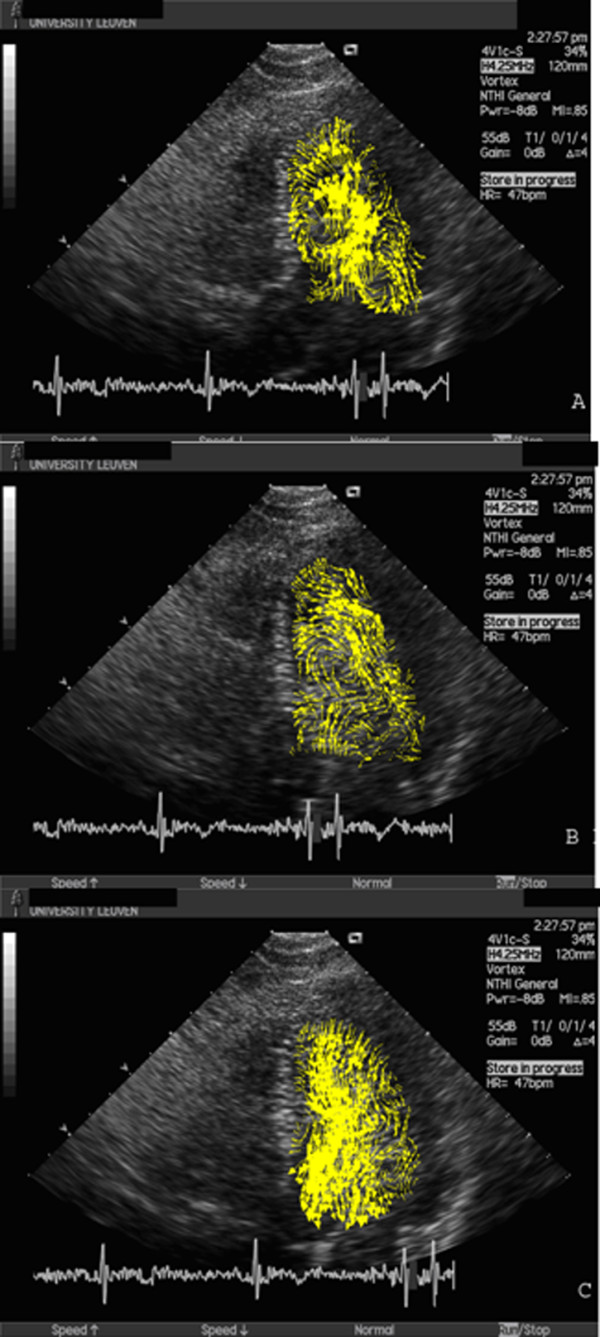
**Sequence analysis of systemic ventricular flow during systole and diastole in Fontan patients and controls**. The vortex from the Fontan group was consistently shorter, wider and rounder than in controls. The vortices were located at the centre of the left ventricle throughout diastole and systole and did not redirect flow in a coherent, sequential fashion as in controls. The location, shape and sphericity of the main vortices differ clearly from controls in all cardiac cycle [early diastole(A), late diastole(B), ejection (C)].

Images of the resulting flow patterns from a 29-year old male Fontan patient, born with DILV, VSD and pulmonary atresia are presented in Figure 1 (Additional file 1). To enable comparison with a normal flow pattern, the flow pattern of a 38-year old male without cardiac abnormalities is depicted in Figure 2 (Additional file 2).

**Figure 3:**
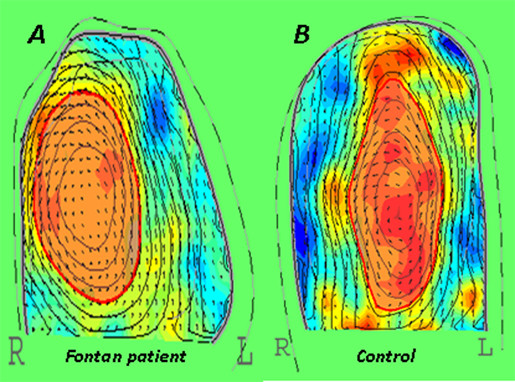
**Colour coded flow data representing the normalized regional vorticity in the plane of the four chamber view (R denotes right; L denotes left), averaged over the cardiac cycle**. Flow data are derived from tracking based echocardiographic particle image velocimetry. Red encodes positive vorticity (i.e. counter clockwise rotation of the blood), blue negative vorticity clockwise rotation). The pulsatility in Fontan group, was less red encodes and was not at the center and the apex of systemic ventricle which means not strong vortex.

In both diastole and systole, distinct and significant differences were observed in the evolution and temporal development of flow structures. The location, shape and sphericity of the main vortices differ clearly from controls in all cardiac cycle [early diastole(A), late diastole(B), ejection (C)] (Figures 1,2). The vortex from the Fontan group was consistently shorter, wider and rounder than in controls. The LV vortex immediately after the onset of the early diastolic phase, redirects the blood flow from LV base, to LV posterior wall, to LV apex during isovolumic relaxation and toward the LV outflow tract and aorta during isovolumic contraction. Pulsatility of systemic ventricular field and vortex represented by RS, VRS and VPC were significantly different in patients with Fontan circulation; red encodes positive vorticity (i.e. counter clockwise rotation of the blood), blue negative vorticity clockwise rotation. The pulsatility in Fontan group was less red encodes and was not at the centre and the apex of systemic ventricle which means not strong vortex. In controls, the vortex was compact, elliptically shaped, and located apically (Figure 3).

### Quantitative analysis of systemic ventricular vortex flow

**Figure 4:**
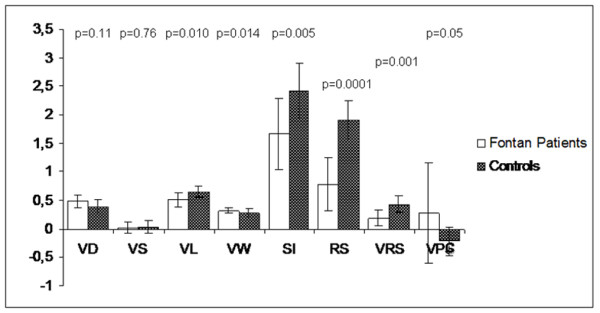
**Comparison of morphological and physiological vortex parameters between controls and the Fontan group**.

VL (0.51 ± 0.09 vs 0.65 ± 0.12, *P *= 0.010), SI (1.66 ± 0.48 vs 2.41 ± 0.42, *p *= 0.005), RS (0.77 ± 0.33 vs 1.90 ± 0.47, *p *= 0.0001), and VRS (0.18 ± 0.13 vs 0.43 ± 0.14, *p *= 0.001) were significantly lower in Fontan patients. VW (0.32 ± 0.006 vs 0.27 ± 0.04, *p *= 0.014) and VPC (0.26 ± 0.25 vs -0.22 ± 0.87, *p *= 0.05) were significantly higher in the Fontan patient group. (Table 3 Figure 4)

**Table 3:** Quantitative Vortex Parameters of Controls and Fontan patients

Variables	Fontan Patients (n = 8)	Controls (n = 15)	*p *value
**VD**	0.484 ± 0.122	0.395 ± 0.111	0.111

**VS**	0.016 ± 0.109	0.031 ± 0.106	0.766

**VL**	0.513 ± 0.097	0.651 ± 0.125	0.010

**VW**	0.320 ± 0.067	0.276 ± 0.044	0.014

**SI**	1.661 ± 0.481	2.412 ± 0.626	0.005

**RS**	0.778 ± 0.334	1.903 ± 0.471	0.0001

**VRS**	0.188 ± 0.134	0.433 ± 0.141	0.001

**VPC**	0.269 ± 0.258	-0.221 ± 0.879	0.050

### Interobserver analysis

Interobserver variability analysis of quantitative vortex parameter measurements are listed in Table 4.

**Table 4:** Interobserver variability analysis of quantitative vortex parameter measurements

Variables	Interobserver variability Mean difference ± 1.96 SD
**VD**	0.012 ± 0.078

**VS**	-0.017 ± 0.056

**VL**	-0.007 ± 0.109

**VW**	-0.010 ± 0.058

**SI**	0.004 ± 0.144

**RS**	-0.003 ± 0.136

**VRS**	0.020 ± 0.044

**VPC**	0.005 ± 0.024

## Discussion

To our knowledge, this is the first report on intracardiac blood flow patterns in Fontan patients using echocardiographic PIV. The PIV technique is noninvasive, and its latest developments allow a high degree of accuracy [8-11]. Shear stress and vortex flow dominate the energetic of any flow; the vortex stores a part of the kinetic energy of the inflow into the rotary motion and redirects it toward the outflow tract [12].

In our study, we were able to achieve reproducible ventricular vortex flow data in patients with Fontan repair and controls by using contrast echocardiography with PIV. The application of PIV to echocardiography seems promising, and results appear to be qualitatively meaningful.

The 8 cases illustrate that structural abnormalities lead to abnormal flow patterns. Adequate vortex formation has been assumed to be energetically important for an optimal cardiac function, because it enables the storing of kinetic energy during diastole which is then subsequently released during systolic ejection. Abnormal geometry and location of the vortex may lead to suboptimal conditions as shown previously in patients with prosthetic mitral valves [6] or left ventricle dysfunction [3,13].

In Fontan patients, diastolic vortex was incoherent, persisted at the centre of ventricle during diastole and systole. Also, this vortex did not dissipate even during systolic ejection, potentially accounting for reduced stroke volume output. The vortex from the Fontan group was consistently shorter, wider and rounder than in controls (Additional files 1 and 2). Pulsatility of systemic ventricular field and vortex represented by RS, VRS and VPC were significantly different in patients with Fontan circulation. There was less red encodes and was not at the centre of ventricle which means not strong vortex. The complexity of the geometry combined with the pulsatile character of the flow, the interaction of the jets with the systemic ventricle flexible walls, and the unsteady motion of the leaflets generate intrinsically complicated turbulent flow structures. So, despite having similar sized systemic ventricle, flow was different in Fontan patients. The clinical implications may be are related to energy loss, adverse flow redirection, high resident times, hemostasis, thrombogenicity, and free emboli formation.

Quantitative vortex parameters may provide a novel method to detect early stages of ventricular dysfunction before gross mechanical changes of ventricular geometry and function as in patients with univentricular heart physiology.

Thus far, heart function has been previously evaluated in echocardiography [14,15] using 2 dimensions measurements (e.g. LVEDd, Simpson EF, etc.), Doppler blood flow patterns, tissue velocity imaging (e.g. E', myocardial acceleration during isovolumic contraction = IVA) and strain analysis. PIV could be an additional method in the future armentarium of the echocardiographist which could be complementary to other methods because it may be helpful to analyze abnormal kinetics in patients with heart failure or those who are prone to heart failure. In addition echocardiographic PIV can contribute to a better understanding of hemodynamic parameters of the heart.

Particularly in Fontan patients, in whom the systemic venous return is re-routed without interference of a subpulmonary ventricle, studies have focused on methods to reduce energy loss in any part of the circulation by optimizing its hemodynamics [16,17]. In this context, it is interesting that recently, a MRI study by Sundareswaran et al., [18] evaluated the value of 3D flow patterns within lateral tunnel and extracardiac Fontan circulations showing large vortices in patients with a lateral tunnel which may also result in increasing power losses. Due to inadequate acoustic windows, imaging of the systemic venous return including vortices was however not possible. MRI and echocardiography may be complementary to each other in these patients

Our data show that location, shape and sphericity of the main vortices differ clearly from controls. We hypothesize that these differences are related to the size and position of the atrioventricular valve, the dimension and morphology of the systemic ventricle, the spatial relationship between ventricular inflow and left ventricular outflow tract (e.g. transposition), or the absence of interference with a functional subpulmonary ventricle. The insights into systemic ventricular vortex flow in Fontan patients may have additional and potentially incremental value over the conventional methods to assess systemic ventricular function. Vortex flow may influence stroke output and efficiency of the systemic ventricular by redirection of intraventricular flow. This has been explored in a preliminary fashion in a previous study [2] but not yet in Fontan patients. Diastolic systemic ventricular vortex characterization may have implication for diastolic volumetric filling and may provide an index that links diastolic filling to systolic stroke volume [19].

After the Fontan procedure a progressive change in systemic ventricular diastolic function is described [20]. Several previous studies have shown impaired systemic ventricular relaxation early after the Fontan procedure [21,22], coincident with the increase in mass: volume ratio and acquired "hypertrophy" of the ventricle after acute preload reduction on transition to the Fontan state [23].

However, in the current era, most patients go through a bidirectional cavopulmonary anastomosis prior to the Fontan procedure, thereby precluding the acute preload reduction that used to occur with transitioning from a shunt to the Fontan. We know that the Fontan circulation involves abnormal loading conditions, with decreased preload reserve and chronically increased afterload, as well as issues with afterload mismatch. This may very well be the reason in this study, for the abnormal vortex flow patterns associated with the single ventricle.

Additional late after the Fontan procedure one previous study have demonstrated changes in diastolic Doppler indices consistent with reduced compliance of the systemic ventricle and persisting abnormalities of relaxation[20]. Diastolic function in children with congenital heart disease has also been characterized using echocardiographic assessment of blood and tissue Doppler velocities, specifically in patients with atrial septal defects, tetralogy of Fallot, single ventricle physiology, and following cardiac transplantation [24].

The PIV and vortices may be a valuable method for the evaluation diastolic and systolic function after the Fontan procedure, but further research is warranted to relate PIV to established measures of cardiac function or symptoms.

Accurate mapping of intraventricular flow provides novel opportunities to evaluate the role of vortices in ventricular function. Similar to any fluid dynamics phenomenon involving vortices, these flow structures are expected to play fundamental roles affecting the dynamics and the energetics of the left ventricle as a pump.

It remains to be determined to what extent these abnormal flow patterns impair systolic and diastolic cardiac function and if surgical corrections aiming at normalizing flow patterns may potentially improve the outcome.

## Limitations

This study included a relatively small number of patients and lacked the correlation to an independent reference such as cardiac catheterization or magnetic resonance imaging (MRI) contrast study.

We decided not to exclude the right ventricle patients because in Fontan patients it is almost not possible to compose a homogeneous population. The complexity (e.g. DILV, tricuspid atresia, Ebstein, valvular disease) and surgical history (e.g. Glenn, BT shunt, Rashkind, etc.) of those patients is often very diverse and every attempt to create a homogeneous dataset will be an illusion. Therefore, we realize that this will always be a limitation in research with patient with CHD.

The current echocardiographic PIV method is 2-dimensional and thus has also limits regarding the analysis of the 3-dimensional structure of flow patterns. This inherent problem of clinical echocardiographic analysis can not be solved because of the low temporal and spatial resolution of the echocardiographic 3-dimensional imaging technology. With the advancements of MRI technology and improvement in the resolution both in time and space of the method, the MRI may be an accurate flow diagnostic method.

Further research is warranted for patients with other forms of cardiomyopathy or congenital heart disease. In addition further research is warranted to compare/contrast this approach with the MRI 4D flow techniques.

## Conclusion

The data from this study showed that it is feasible to quantify systemic ventricular vortex flow using contrast vector profile in Fontan patients. Vortex characteristics differ significantly from normal controls. This motivates for further research to assess the clinical implications where necessary.

## Abbreviations

ASD: Atrial septal defect; AVSD: Atrioventricular septal defect; DILV: Double inlet left ventricle; DORV: Double outlet right ventricle; EF: Ejection fraction; FS: Fractional shortening; LAd: Left atrial dimension; LVEDd: Left ventricular end diastolic dimension; LVESd: Left ventricular end systolic dimension; MRI: Magnetic resonance imaging: PS: Pulmonary stenosis; RS: Relative strength; SI: Sphericity index; SVC: Superior vena cava; TA: Tricuspid atresia; cc-TGA: Congenitally corrected transposition of the great arteries; TS: Tricuspid stenosis; VD: Vortex depth; VL: Vortex length; VPC: Vortex pulsation correlation; PIV: Practicle image velocimetry; VRS: Vortex relative strength; VS: Vortex strength; VSD: Ventricular septal defect; VW: Vortex width

## Competing interests

The authors declare that they have no competing interests.

## Authors' contributions

WB, JP van M, ET, in the data collection. KL, AVan de B, in the laboratory analysis, and in statistical analysis. KL, JP van M, WB, in the interpretation of data and manuscript preparation. All authors read and approved the final Manuscript.

## Supplementary Material

Additional file 1 **The flow patterns from a 29-year old male Fontan patient**. Click here for file

Additional file 2 **The flow pattern of a 38-year old male without cardiac abnormalities**. Click here for file

## References

[B1] Zheng H Mukdadi O Hertzberg J Shandas R Advantages in using multi-frequency driving ultrasound for optimizing echo particle image velocimetry techniques Biomed Sci Instrum 2004 40 371 376 15133986

[B2] Sengupta PP Khandheria BK Korinek J Jahangir A Yoshifuku S Milosevic I Belohlavek M Left ventricular isovolumic flow sequence during sinus and paced rhythms: new insights from use of high-resolution Doppler and ultrasonic digital particle imaging velocimetry J Am Coll Cardiol 2007 49 899 908 10.1016/j.jacc.2006.07.075 17320749

[B3] Hong GR Pedrizzetti G Tonti G Li P Wei Z Kim JK Baweja A Liu S Chung N Houle H Narula J Vannan MA Characterization and quantification of vortex flow in the human left ventricle by contrast echocardiography using vector particle image velocimetry JACC Cardiovasc Imaging 2008 1 705 717 10.1016/j.jcmg.2008.06.008 19356506 PMC4695212

[B4] Sengupta PP Burke R Khandheria BK Belohlavek M Following the flow in chambers Heart Fail Clin 2008 4 325 332 10.1016/j.hfc.2008.02.005 18598984

[B5] Kilner PJ Yang GZ Wilkes AJ Mohiaddin RH Firmin DN Yacoub MH Asymmetric redirection of flow through the heart Nature 2000 404 759 761 10.1038/35008075 10783888

[B6] Faludi R Szulik M D'hooge J Herijgers P Rademakers F Pedrizzetti G Voigt JU Left ventricular flow patterns in healthy subjects and patients with prosthetic mitral valves. An *in-viv*-study using Echocardiographic Particle Image Velocimetry J Thorac Cardiovasc Surg 2010 139 1501 1510 10.1016/j.jtcvs.2009.07.060 20363003

[B7] Bland JM Altman DG Measuring agreement in method comparison studies Stat Methods Med Res 1999 8 135 160 10.1191/096228099673819272 10501650

[B8] Cenedese A Del Prete Z Miozzi M Querzoli G A laboratory investigation of the flow in the left ventricle of the human heart with prosthetic, tilting-disk valves Exp Fluid 2005 39 322 335 10.1007/s00348-005-1006-4

[B9] Kim HB Hertzberg JR Shandas R Development and validation of echo PIV Exp Fluid 2004 36 455 462 10.1007/s00348-003-0743-5

[B10] Mukdadi OM Kim HB Hertzberg J Shandas R Numerical modelling of microbubble backscatter to optimize ultrasound particle image velocimetry imaging Ultrasonics 2004 42 1111 1121 10.1016/j.ultras.2004.02.021 15234173

[B11] Domenichini F Querzoli G Cenedese A Pedrizzetti G Combined experimental and numerical analysis of the flow structure into the left ventricle J Biomech 2007 40 1988 1994 10.1016/j.jbiomech.2006.09.024 17097665

[B12] Kheradvar A Houle H Pedrizzetti G Tonti G Belcik T Ashraf M Lindner JR Gharib M Sahn D Echocardiographic Particle Image Velocimetry: A Novel Technique for Quantification of Left Ventricular Blood Vorticity Pattern J Am Soc Echocardiogr 2010 23 86 94 10.1016/j.echo.2009.09.007 19836203

[B13] Mohiaddin RH Flow patterns in the dilated ischemic left ventricle studied by MR imaging with velocity vector mapping J Magn Reson Imaging 1995 5 493 498 10.1002/jmri.1880050503 8574031

[B14] Lang RM Bierig M Devereux RB Flachskampf FA Foster E Pellikka PA Picard MH Roman MJ Seward J Shanewise J Solomon S Spencer KT St John Sutton M Stewart W American Society of Echocardiography's Nomenclature and Standards Committee; Task Force on Chamber Quantification; American College of Cardiology Echocardiography Committee; American Heart Association; European Association of Echocardiography European Society of Cardiology Eur J Echocardiogr 2006 2 79 108

[B15] Mor-Avi V Lang RM Badano LP Belohlavek M Cardim NM Derumeaux G Galderisi M Marwick T Nagueh SF Sengupta PP Sicari R Smiseth OA Smulevitz B Takeuchi M Thomas JD Vannan M Voigt JU Zamorano JL Current and evolving echocardiographic techniques for the quantitative evaluation of cardiac mechanics: ASE/EAE consensus statement on methodology and indications endorsed by the Japanese Society of Echocardiography Eur J Echocardiogr 2011 12 3 167 205 10.1093/ejechocard/jer021 21385887

[B16] Huddleston CB The failing Fontan: options for surgical therapy Pediatr Cardiol 2007 28 472 476 10.1007/s00246-007-9008-z 17955283

[B17] Mavroudis C Backer CL Deal BJ Late reoperations for Fontan patients: state of the art invited review Eur J Cardiothorac Surg 2008 34 1034 1040 10.1016/j.ejcts.2008.04.024 18977665

[B18] Sundareswaran KS Haggerty CM de Zélicourt D Dasi LP Pekkan K Frakes DH Powell AJ Kanter KR Fogel MA Yoganathan AP Visualization of flow structures in Fontan patients using 3-dimensional phase contrast magnetic resonance imaging J Thorac Cardiovasc Surg 2011 in press PubMed PMID: 22088274 10.1016/j.jtcvs.2011.09.067 PMC3437662 22088274

[B19] Le Jemtel TH Alt EU Are hemodynamic goals viable in tailoring heart failure therapy? Hemodynamic goals are outdated Circulation 2006 113 1027 1032 16493785

[B20] Cheung YF Penny DJ Redington AN Serial assessment of left ventricular diastolic function after Fontan procedure Heart 2000 83 420 424 10.1136/heart.83.4.420 10722541 PMC1729362

[B21] Frommelt PC Snider AR Meliones JN Vermilion RP Doppler assessment of pulmonary artery flow patterns and ventricular function after the Fontan operation Am J Cardiol 1991 68 1211 1215 10.1016/0002-9149(91)90195-Q 1951081

[B22] Akagi T Benson LN Gilday DL Ash J Green M Williams WG Freedom RM Influence of ventricular morphology on diastolic filling performance in double-inlet ventricle after the Fontan procedure J Am Coll Cardiol 1993 22 1948 1952 10.1016/0735-1097(93)90784-X 8245354

[B23] Penny DJ Redington AN Angiographic demonstration of incoordinate motion of the ventricular wall after the Fontan operation Br Heart J 1991 66 456 459 10.1136/hrt.66.6.456 1772713 PMC1024822

[B24] Frommelt PC Echocardiographic measures of diastolic function in pediatric heart disease Curr Opin Cardiol 2006 21 3 194 199 10.1097/01.hco.0000221580.63996.93 16601456

