# Correction: Visualization of the intracavitary blood flow in systemic ventricles of Fontan patients by contrast echocardiography using particle image velocimetry

**DOI:** 10.1186/1476-7120-10-18

**Published:** 2012-04-26

**Authors:** Konstantinos Lampropoulos, Werner Budts, Alexander Van de Bruaene, Els Troost, Joost P van Melle

**Affiliations:** 1 Department of Cardiology, University Hospitals Leuven, Leuven, Belgium; 2 Department of Cardiology, Polyclinic General Hospital of Athens, Athens, Greece; 3 Department of Cardiology, University Medical Center Groningen, University of Groningen, Groningen, The Netherlands; 4 Congenital and Structural Cardiology, University Hospitals Leuven, Herestraat 49, 3000, Leuven, Belgium

## 

Following publication of our article [1] the authors noted that the legends for Figure 1 and Figure 2 were incorrect.

The correct legend for Figure 1 is:

**Sequence analysis of systemic ventricular flow during systole and diastole in Fontan patients.** The vortex from the Fontan group was consistently shorter, wider and rounder. The vortices were located at the centre of the left ventricle throughout diastole and systole and did not redirect flow in a coherent, sequential fashion as in controls. The location, shape and sphericity of the main vortices differ clearly from controls in all cardiac cycle [early diastole(A), late diastole(B), ejection (C)].

The correct legend for Figure 2 is:

**Sequence analysis of systemic ventricular flow during systole and diastole in controls.** The vortex from the control group was compact, elliptically shaped, and located apically. The location, shape and sphericity of the main vortices differ clearly from the Fontan group in all cardiac cycle [early diastole(A), late diastole(B), ejection (C)].

It was also noted the legends for the Additional file 1 and Addition file 2 were also incorrect:

The correct legend for Additional file 1 is:

The flow patterns of a 38 year old female without cardiac abnormalities

The correct legend for Additional file 2 is:

The flow pattern of a 29 year old male with Fontan circulation.

The authors would like to apologize for any inconvenience caused by this error.
